# Anti-Inflammatory Effects and Mechanisms of Action of Coussaric and Betulinic Acids Isolated from *Diospyros kaki* in Lipopolysaccharide-Stimulated RAW 264.7 Macrophages

**DOI:** 10.3390/molecules21091206

**Published:** 2016-09-09

**Authors:** Kyoung-Su Kim, Dong-Sung Lee, Dong-Cheol Kim, Chi-Su Yoon, Wonmin Ko, Hyuncheol Oh, Youn-Chul Kim

**Affiliations:** 1 Research Institute of Pharmaceutical Sciences, Keimyung University, 1095 Dalgubeol-Daero, Daegu 42601, Korea; kskim@kmu.ac.kr; 2 College of Pharmacy, Chosun University, Dong-Gu, Gwangju 61452, Korea; dslee2771@chosun.ac.kr; 3 Institute of Pharmaceutical Research and Development, College of Pharmacy, Wonkwang University, Iksan 54538, Korea; kimman07@hanmail.net (D.-C.K.); ycs1991@naver.com (C.-S.Y.); rabis815@naver.com (W.K.); hoh@wku.ac.kr (H.O.)

**Keywords:** *Diospyros kaki* Thunb., coussaric acid (CA), betulinic acid (BA), anti-inflammation, nuclear factor-kappa B, heme oxygenase-1

## Abstract

*Diospyros kaki* Thunb. is widely distributed in East Asian countries, its leaves being mainly used for making tea. In this study, coussaric acid (CA) and betulinic acid (BA), both triterpenoid compounds, were obtained from *D. kaki* leaf extracts through bioassay-guided isolation. CA and BA showed anti-inflammatory effects via inhibition of the nuclear factor-κB (NF-κB) pathway, providing important information on their anti-inflammatory mechanism. Furthermore, they markedly inhibited nitric oxide (NO) and prostaglandin E_2_ (PGE_2_) production in lipopolysaccharide (LPS)-activated RAW 264.7 macrophages, and suppressed tumor necrosis factor-α (TNF-α), interleukin-6 (IL-6), and interleukin-1β (IL-1β) levels. Furthermore, they decreased protein expression of inducible nitric oxide synthase and cyclooxygenase-2. Pre-treatment with CA and BA inhibited LPS-induced NF-κB. We further examined the effects of CA and BA on heme oxygenase (HO)-1 expression in RAW 264.7 macrophages: BA induced HO-1 protein expression in a dose-dependent manner, while CA had no effect. We also investigated whether BA treatment induced nuclear translocation of Nrf2. BA inhibited LPS-induced NF-κB-binding activity, as well as pro-inflammatory mediator and cytokine production (e.g., NO, PGE_2_, TNF-α, IL-1β, IL-6), by partial reversal of this effect by SnPP, an inhibitor of HO-1. These findings further elucidate the anti-inflammatory mechanism of CA and BA isolated from *D. kaki*.

## 1. Introduction

Inflammation is an important part of the protective immune response against harmful stimuli. However, uncontrolled inflammation can lead to the development of diseases, such as inflammatory bowel disease, rheumatoid arthritis, neurodegenerative disorders, and sepsis [1]. Lipopolysaccharide (LPS), an exogenous bacterial endotoxin, activates macrophages such that they produce various pro-inflammatory cytokines and mediators, including tumor necrosis factor-α (TNF-α), interleukin-1β (IL-1β), interleukin-6 (IL-6), nitric oxide (NO), and prostaglandin E_2_ (PGE_2_) [2,3]. Nuclear factor-κB (NF-κB) is a key transcriptional factor involved in immune and inflammatory responses [4]. In the inactive state, NF-κB exists in the cytoplasm and complexes with the inhibitor of NF-κB (IκB). Heme oxygenase-1 (HO-1) has been recognized as an important molecule in the regulation of inflammation because it inhibits the production of pro-inflammatory cytokines and mediators in activated macrophages [5,6,7]. Nuclear transcription factor-E2-related factor 2 (Nrf2) has been reported to be crucial for HO-1 induction [8]. 

Recently, traditional herbal medicines have provided an interesting potential source for new drugs in modern medicine [9,10]. *Diospyros kaki* Thunb. (Ebenaceae) is widely cultivated in East Asian countries, and its leaves are commonly used for making tea. It has been widely used in Asia for the treatment of various diseases, such as atherosclerosis, ischemia, and hypotension [11,12,13,14,15]. Previous reports indicate that the beneficial components of *D. kaki* include flavonoids, tannins, triterpenoids, and vitamin A [16]. Triterpenoids in *D. kaki* have various biological activities, such as anti-oxidant [17], anti-diabetic [18], and anti-tumor [19] effects. Thus, a detailed study on these triterpenoids would prove significant and valuable for human health. Betulinic acid (BA), a pentacyclic triterpene, has been reported to have various biological activities, such as anti-tumor [20], anti-inflammatory [21,22], and anti-malarial [23] effects. However, the relationship between BA and HO-1 expression is still unclear. Moreover, the biological activity of coussaric acid (CA) has not yet been elucidated. 

In this study, we demonstrated the inhibitory effect of a 70% EtOH extract of *D. kaki* leaves (DKLE) on the inflammatory reaction in LPS-stimulated RAW 264.7 macrophages. We also obtained CA and BA from DKLE through bioassay-guided isolation, and examined their anti-inflammatory effects.

## 2. Results

### 2.1. Isolation of CA and BA

**Figure 1:**
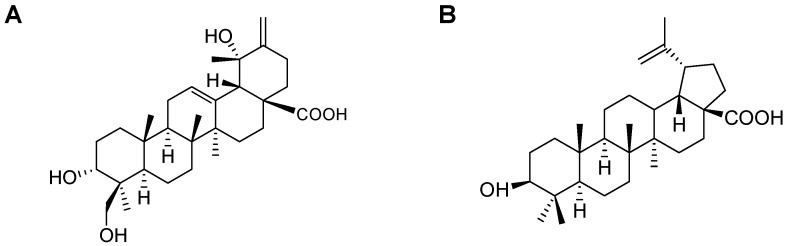
Structures of (**A**) coussaric acid (CA) and (**B**) betulinic acid (BA).

By using the NO assay, we tested to isolate the compounds with anti-inflammatory properties from fractions 4 and 5, and obtained CA and BA (Figure 1). The structures of CA and BA were identified by proton nuclear magnetic resonance (^1^H-NMR), ^13^C-NMR, and distortionless enhancement by polarization transfer (DEPT), and confirmed by comparing the NMR spectral data with those previously reported [24,25,26,27,28]. The ^1^H-NMR and ^13^C-NMR spectral data of CA and BA are listed in Table 1 and Table 2. CA was obtained as colorless needles, with a melting point (mp) of 232–233 °C and molecular formula C_30_H_46_O_5_ (calc. for C_30_H_46_O_5_, 486.3345). BA was obtained as a white powder, with an mp of 295–298 °C and molecular formula C_30_H_48_O_3_ (calc. for C_30_H_48_O_3_, 456.7003). The relationship between BA and HO-1 expression is unknown. In addition, the biological activity of CA, a triterpenoid with an ursane skeleton, is unknown.

### 2.2. Inhibitory Effects of CA and BA on the Production of Pro-Inflammatory Mediators and Enzymes in LPS-stimulated RAW 264.7 Macrophages

**Figure 2:**
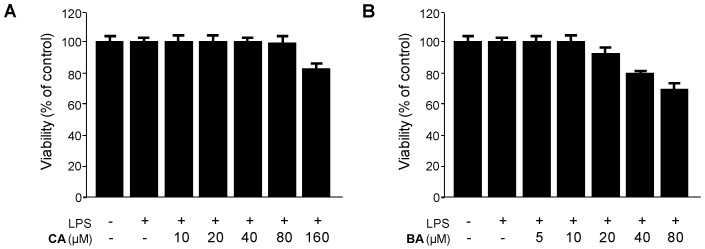
Effects of (**A**) CA and (**B**) BA on cell viability of RAW 264.7 macrophages stimulated with LPS. (**A**,**B**) Cells were incubated for 24 h with the indicated concentrations of CA and BA. Cell viability was determined as described in the Materials and Methods. Data shown represent the mean values of three experiments ±SD.

Cytotoxic effects of CA and BA on RAW 264.7 macrophages were determined by using the tetrazolium salt 3-[4,5-dimethylthiazol-2-yl]-2,5-diphenyltetrazolium bromide (MTT) assay. The viability of cells incubated with different concentrations of CA (10–160 μM) and BA (5–80 μM) was not affected at concentrations up to 80 μM and 10 μM (Figure 2). Subsequent experiments were conducted at non-toxic concentrations of CA and BA.

We evaluated the inhibitory effects of CA and BA on NO and PGE_2_ production in RAW 264.7 macrophages. RAW 264.7 macrophages were treated with the indicated concentrations of CA and BA for 3 h prior to LPS treatment for 24 h.

**Figure 3:**
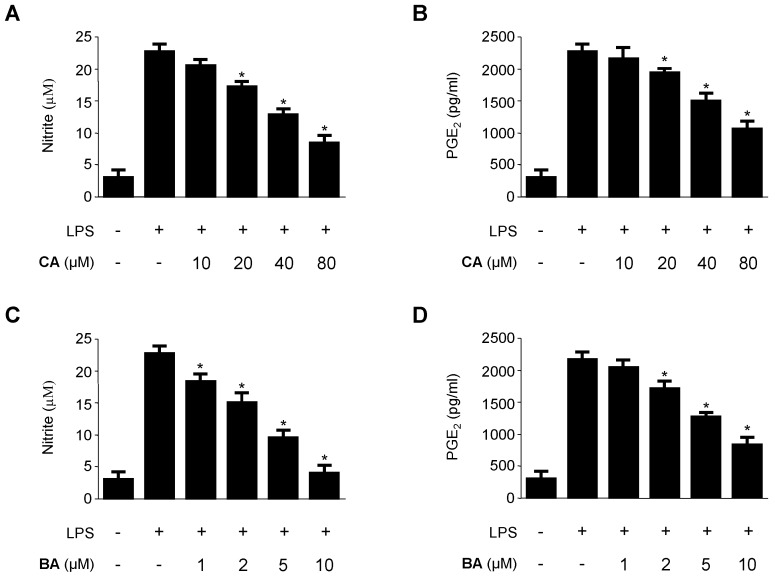
Effects of CA and BA on the production of (**A**) nitrite and (**B**) PGE_2_ of RAW 264.7 macrophages stimulated with LPS. (**A**,**B**) The cells were pre-treated with indicated concentrations of CA and BA for 12 h, and then stimulated with LPS (1 μg/mL) for 18 h. The production of nitrite and PGE_2_ was determined as described in the Materials and Methods. Data shown represent the mean values of three experiments ±SD. * *p* < 0.05 as compared to the group treated with LPS alone.

**Figure 4:**
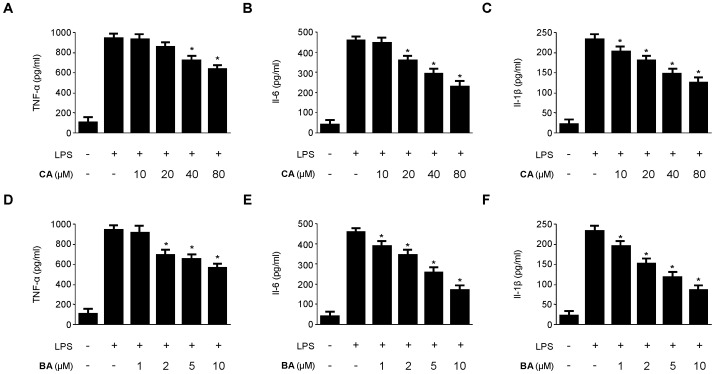
Effects of CA and BA on the production of (**A**,**D**) TNF-α; (**B**,**E**) IL-6; and (**C**,**F**) IL-1β in RAW 264.7 macrophages stimulated with LPS. (**A**–**F**) Cells were pre-treated with indicated concentrations of CA and BA for 3 h, and then stimulated with LPS (1 μg/mL) for 24 h. Production of TNF-α, IL-1β, and IL-6 was measured as described in the Materials and Methods. Data shown represent the mean values of three experiments ±SD. * *p* < 0.05 as compared with the group treated with LPS alone.

As shown in Figure 3, CA and BA markedly inhibited the production of NO and PGE_2_ in LPS-activated RAW 264.7 macrophages in a dose-dependent manner. To confirm the effect of CA and BA on the production of pro-inflammatory cytokines, such as TNF-α, IL-6, and IL-1β, cells were stimulated with LPS (1 μg/mL) for 24 h in the presence or absence of non-cytotoxic concentrations of CA and BA. As shown in Figure 4, CA and BA suppressed the levels of TNF-α, IL-6, and IL-1β in a dose-dependent manner, as measured by ELISA.

### 2.3. Effects of CA and BA on iNOS and COX-2 Expression and NF-κB Activation in LPS-Stimulated RAW 264.7 Macrophages

**Figure 5:**
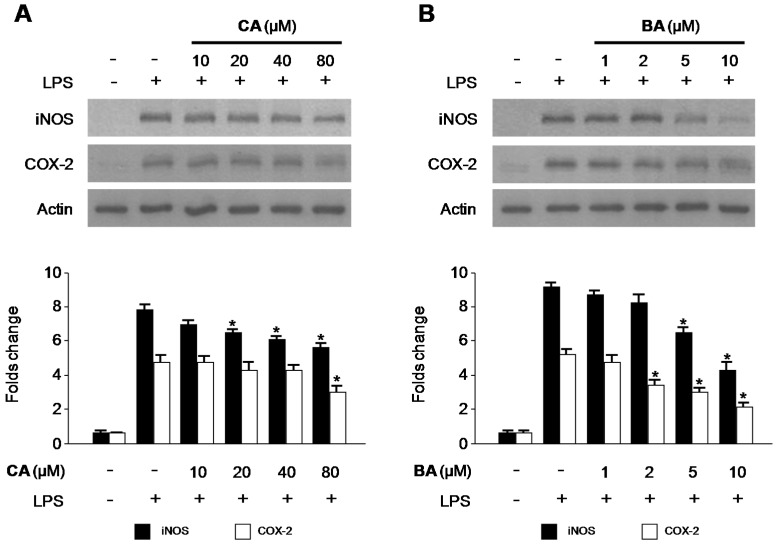
Effects of CA and BA on (**A**) iNOS and (**B**) COX-2 protein expression in RAW 264.7 macrophages stimulated with LPS. (**A**,**B**) Cells were pre-treated with indicated concentrations of CA and BA for 3 h, and then stimulated with LPS (1 μg/mL) for 24 h. Western blot analysis was performed as described in the Materials and Methods, and representative blots from three independent experiments that showed similar results were chosen. Data shown represent the mean values of three experiments ± SD. * *p* < 0.05 as compared with the group treated with LPS alone.

**Figure 6:**
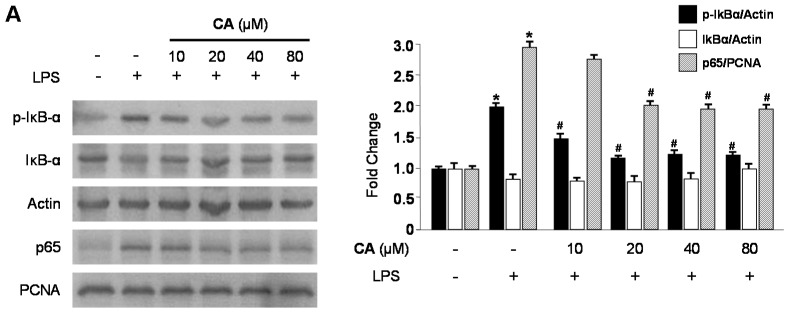

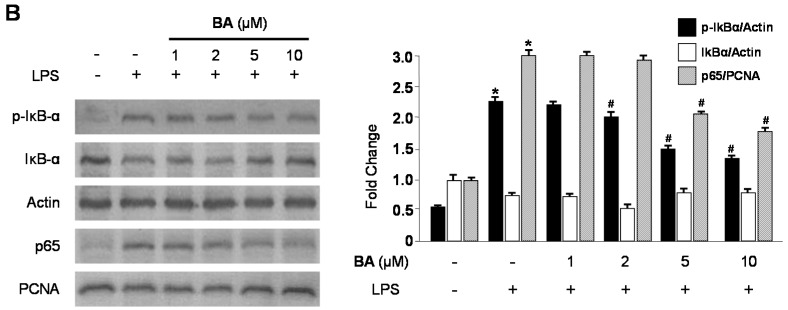
Effects of CA and BA on (**A**,**B**) NF-κB activation. (**A**,**B**) Cells were pre-treated with the indicated concentrations of CA and BA for 3 h, and then stimulated with LPS (1 μg/mL) for 30 min. Western blot analysis was performed as described in the Materials and Methods, and representative blots from three independent experiments that showed similar results were chosen. * *p* < 0.05 as compared with the control group. # *p* < 0.05 as compared with the group treated with LPS alone.

We investigated the effects of CA and BA on LPS-induced inducible nitric oxide synthase (iNOS) and COX-2 protein upregulation in RAW 264.7 macrophages. Cells were treated with the indicated concentrations of CA and BA for 3 h prior to LPS (1 μg/mL) treatment for 24 h, and the expression of iNOS and COX-2 were measured. As shown in Figure 5, CA and BA decreased the protein expression of iNOS and COX-2, in a dose-dependent manner. We tested to determine whether CA and BA inhibit the phosphorylation and degradation of IκB-α, and the translocation of NF-κB (p65) into the nucleus. As shown in Figure 6, IκB-α was degraded and p65 was translocated after treatment with LPS (30 min) in RAW 264.7 macrophages. However, LPS-induced NF-κB activation was significantly inhibited through pre-treatment with various concentrations of CA and BA for 3 h, in a dose-dependent manner.

### 2.4. Effects of CA and BA on HO-1 Expression and Nrf2 Nuclear Translocation in RAW 264.7 Macrophages

**Figure 7:**
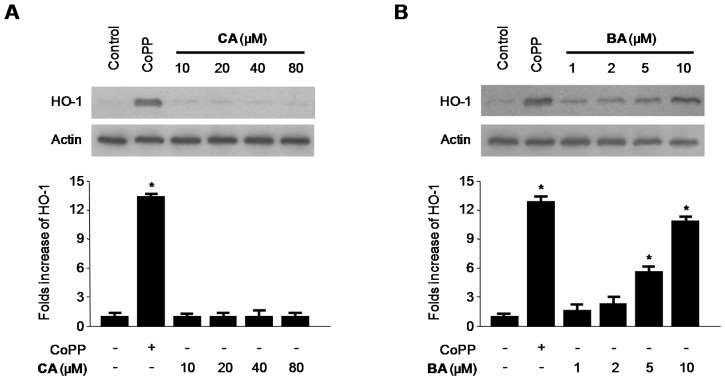
Effects of CA and BA on (**A**,**B**) HO-1 expression in RAW 264.7 macrophages. (**A**,**B**) Cells were incubated for 12 h with the indicated concentrations of CA, BA, and CoPP (20 μM), a HO-1 Inducer, was used as the positive control. Western blot analysis was performed as described in the Materials and Methods, and representative blots from three independent experiments that showed similar results were chosen. Data shown represent the mean values of three experiments ± SD. * *p* < 0.05 as compared with the control.

We examined the effects of CA and BA on HO-1 expression in RAW 264.7 macrophages. Various concentrations of BA induced HO-1 protein expression in a dose-dependent manner in cells treated for 12 h (Figure 7B). In contrast, CA had no effect on the expression of HO-1. Accordingly, we investigated whether BA-induced HO-1 expression is associated with the nuclear translocation of Nrf2. Because Nrf2 plays a crucial role in the transcriptional activation of HO-1 gene expression [29], we specifically investigated whether treatment with BA induces the nuclear translocation of Nrf2.

**Figure 8:**
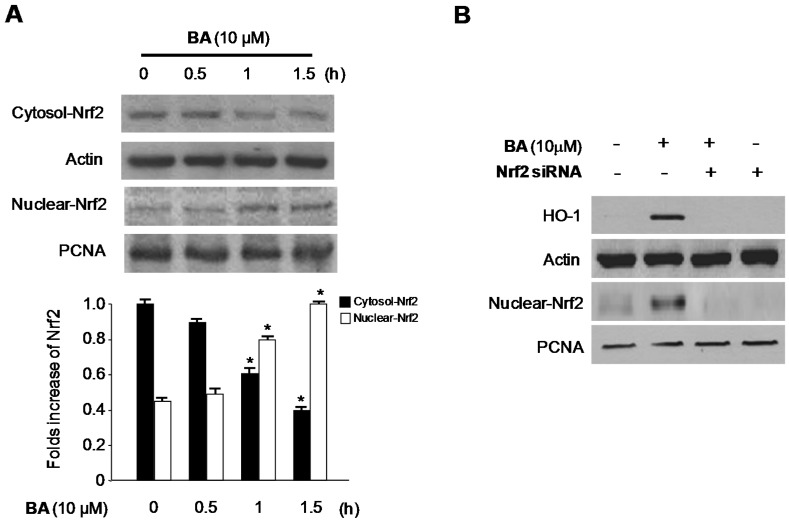
Effects of BA on the nuclear translocation of (**A**) Nrf2 and (**B**) Nrf2-mediated HO-1 in RAW 264.7 macrophages. (**A**) Cells were treated for the indicated periods with 10 μM BA. Nuclei were fractionated from the cytosol using PER-Mammalian Protein Extraction buffer, as described in the materials and methods; (**B**) RAW 264.7 macrophages were transiently transfected with Nrf2 siRNA and then treated with 10 μM BA for 12 h. Transfection and western blot analysis was performed as described in the Materials and Methods. Data shown represent the mean values of three experiments ±SD. * *p* < 0.05 as compared with the control.

Cells incubated with 10 μM BA for 0.5, 1, and 1.5 h showed increased nuclear Nrf2 levels and decreased cytoplasmic Nrf2 levels (Figure 8A). In addition, the role of Nrf2 in BA-induced HO-1 expression was studied using a siRNA against Nrf2. RAW 264.7 macrophages were transiently transfected with Nrf2 siRNA, and then treated with 10 μM BA for 12 h. As shown in Figure 8B, transient transfection with Nrf2 siRNA completely abolished BA-induced HO-1 expression.

### 2.5. Effects of HO-1 Expression on the Inhibition of Pro-Inflammatory Mediators, Cytokines, and NF-κB Activity by BA in LPS-Stimulated RAW 264.7 Macrophages

**Figure 9:**
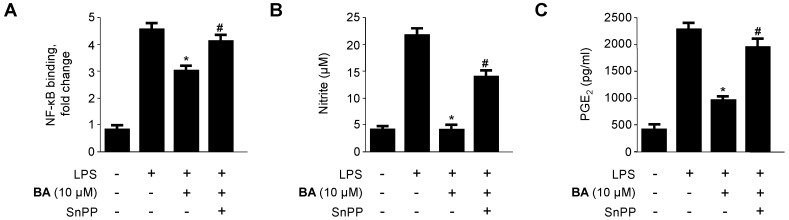

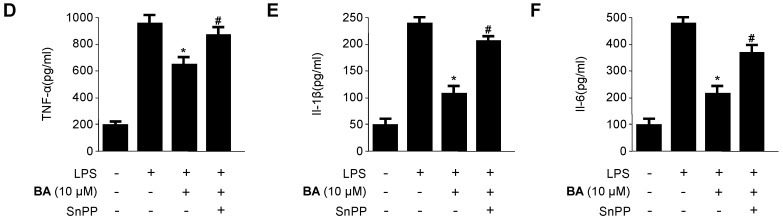
Effects of SnPP on BA-mediated inhibition of (**A**–**F**) NF-κB activation and nitrite, PGE_2_, TNF-α, IL-1β, and IL-6 production in LPS-stimulated RAW 264.7 macrophages. Cells were pre-treated with BA for 3 h in the presence or absence of SnPP (50 μM), and then stimulated with LPS (1 μg/mL) for (**A**) 30 min or (**B**–**F**) 24 h. Production of nitrite, PGE_2_, TNF-α, IL-1β, and IL-6 and the degree of NF-κB binding were determined as described in Materials and Methods. Data shown represent the mean values of three experiments ± SD. * *p* < 0.05 compared with the group treated with LPS alone; # *p* < 0.05 compared with the group treated with BA and LPS.

To confirm that the anti-inflammatory effect of BA correlated with HO-1 expression via the Nrf2 pathway, we investigated whether the effect of BA-induced HO-1 expression could be reversed by pre-treatment with SnPP, an inhibitor of HO-1. RAW 264.7 macrophages were pre-treated with 10 μM BA for 3 h in the absence or presence of SnPP, followed by LPS stimulation for 24 h. As shown in Figure 9, the inhibitory effects of BA toward LPS-induced NF-κB-binding activity and pro-inflammatory mediator and cytokine production (e.g., NO, PGE_2_, TNF-α, IL-1β, and IL-6) were partially reversed by SnPP.

## 3. Discussion

Medicinal plants have become an essential part of health care, based on increased scientific research [9,10]. Recently, various studies have reported that *D. kaki* Thunb. (Ebenaceae) has anti-inflammatory [30] and anti-oxidant [31] effects. We tested to isolate CA and BA from fractions of DKLE by NO production, as they have anti-inflammatory properties. CA was obtained as colorless needles with molecular formula C_30_H_46_O_5_, and BA was obtained as a white powder with molecular formula C_30_H_48_O_3_ (Figure 1). The biological activity of CA or BA is still mostly unknown. Therefore, we investigated the anti-inflammatory effects and mechanisms of CA and BA in LPS-stimulated RAW 264.7 macrophages.

Macrophages are critical cells in the development of inflammatory reactions, as they excessively produce or secrete various pro-inflammatory mediators and cytokines [29,30,32]. NO plays an important role in inflammatory response as a pro-inflammatory molecule, which is produced by iNOS. Uncontrolled or excess NO production leads to the development of various inflammatory diseases [33,34,35]. Therefore, inhibition of iNOS and NO expression was assessed for anti-inflammatory potential. We investigated whether BA and CA blocked the production of NO and iNOS protein expression in LPS-stimulated inflammatory condition in RAW 264.7 macrophages (Figure 3A,C and Figure 5). Cyclooxygenase-2 (COX-2) is involved in the synthesis of PGE_2_, which produces inflammatory symptoms, including fever and pain [34,35,36]. A number of anti-inflammatory drugs target the suppression of PGE_2_ production and COX-2 expression. TNF-α, IL-1β, and IL-6 play a key role in triggering and promoting inflammation in macrophages [36]. Therefore, suppression of pro-inflammatory cytokines and mediators is vital to control immune responses. We investigated whether BA and CA, components of *D. kaki* Thunb. (Ebenaceae), blocked the production of pro-inflammatory cytokines in LPS-induced inflammatory RAW 264.7 macrophages. BA and CA also suppressed the levels of COX-2, and the mRNA level of various pro-inflammatory cytokines, including TNF-α, IL-1β, IL-6, and IL-12 (Figure 4 and Figure 5). These all findings suggest that BA and CA, at least in LPS-stimulated RAW 264.7 macrophages, exert their anti-inflammatory effects by limiting the expression of pro-inflammatory enzymes and cytokines.

Nuclear factor-κB (NF-κB) is an important transcriptional factor involved in inflammation. Upon activation by external stimuli such as TNF-α and LPS, the IκB protein is phosphorylated and degraded, leading to its translocation into the nucleus [37]. Translocated NF-κB interacts with κB elements in the promoter region of various inflammatory genes, leading to the transcription of pro-inflammatory mediators and cytokines including iNOS, COX-2, NO, PGE_2_, TNF-α, IL-6, and IL-1β [38,39]. Thus, NF-κB has been regarded as the molecular target in development of therapies for inflammatory diseases [40]. In this study, we examined the inhibitory effects of BA and CA on NF-κB, p50, and p65 translocation, and IκBα phosphorylation and degradation. Following treatment with BA and CA, LPS-induced NF-κB activation and IκBα degradation were inhibited in RAW 264.7 macrophages (Figure 6). Accordingly, the inhibition of the NF-κB pathway in RAW 264.7 macrophages by BA and CA down-regulated the pro-inflammatory mediators, existing an anti-inflammatory effect.

HO-1 is an inducible rate-limiting enzyme involved in heme catabolism, converting heme to biliverdin, ferrous iron, and carbon monoxide (CO) [41]. Under normal conditions, Nrf2 is complexed with the negative regulator of Nrf2, Kelch-like ECH-associated protein (Keap1) in the cytosol [42]. This complex is disrupted under stressful cellular conditions; Nrf2 separates from Keap1 and translocates into the nucleus, where it binds to the antioxidant response element (ARE), a regulatory element in the promoter regions of phase II enzymes, including HO-1 [43]. In this study, we examined the induction of HO-1 after treatment with BA and CA. HO-1 protein expression increased dose-dependently after treatment with BA, but not CA (Figure 7). In addition, BA also increased the Nrf2 translocation time-dependently (Figure 8A). Moreover, we investigated HO-1 protein expression after treatment with BA and Nrf2 siRNA. When BA is treated with Nrf2 siRNA simultaneously, HO-1 expression is inhibited (Figure 8B). As shown in Figure 8B, transient transfection with Nrf2 siRNA completely abolished HO-1 expression by BA, which suggested that BA was associated with HO-1 expression via Nrf2 signaling pathways. Furthermore, the inhibitory effects of BA on the production of inflammatory cytokines in LPS-treated RAW 264.7 macrophages were partially reversed by treatment with SnPP, an inhibitor of HO-1 enzyme activity (Figure 9). These results suggest that the induction of HO-1 is involved in the inhibitory effects of BA on the production of pro-inflammatory mediators and cytokines via the NF-κB pathway. On the other hand, CA has anti-inflammatory action through only NF-κB pathway, but not HO-1/Nrf2 related pathways. 

## 4. Materials and Methods

### 4.1. General Information

NMR spectra were recorded in pyridine by using a JNM ECP-400 spectrometer operating (JEOL, Peabody, MA, USA) at 400 MHz for ^1^H and at 100 MHz for ^13^C. Flash column chromatography was performed using octadecyl-functionalized silica gel C_18_ (12 nm, S-75 μm, YMC, Kyoto, Japan). TLC was carried out on silica gel 60 F_254_ plates (Merck, Darmstadt, Germany). Dulbecco’s modified Eagle’s medium, fetal bovine serum, and other tissue culture reagents were purchased from Gibco BRL Co. (Grand Island, NY, USA). All chemicals were obtained from Sigma Chemical Co. (St. Louis, MO, USA). Small interfering RNA (siRNA) for Nrf2 and antibodies to iNOS, COX-2, phosphor (p)-IκBα, IκBα, p65, PCNA, and actin were obtained from Santa Cruz Biotechnology (Santa Cruz, CA, USA). HO-1 and Nrf2 antibodies were obtained from Cell Signaling Technology (Cell Signaling, Danvers, MA, USA). Tin protoporphyrin IX (SnPP), an inhibitor of HO activity, was obtained from Porphyrin Products (Logan, UT, USA). Enzyme-linked immunosorbent assay (ELISA) kits for PGE_2_, TNF-α, IL-1β, and IL-6 were purchased from R&D Systems, Inc. (Minneapolis, MN, USA).

### 4.2. Sample Preparation

*D. kaki* Thunb. (Ebenaceae) leaves were obtained from the botanical garden of Wonkwang University, Iksan, Korea, in August 2012. The voucher specimen (WK-2012-08-23) was deposited at the Herbarium of the College of Pharmacy, Wonkwang University (Korea). Dried leaves of *D. kaki* (114.32 g) were subjected to extraction with 70% EtOH in H_2_O (3 L) by boiling for 2 h. The 70% EtOH extract (31.31 g) was obtained, and some of the extract (5.13 g) was dissolved in MeOH. Continually, the extract (5.13 g) was subjected to C_18_-functionalized silica gel open column chromatography and eluted with a stepwise gradient of 20%, 40%, 60%, 80%, and 100% (*v/v*) of MeOH in H_2_O (500 mL each). The fraction (101.1 mg) eluted with 80% MeOH was subjected to a silica gel column chromatography (2.7 × 57 cm) by using a gradient elution (CH_2_Cl_2_:MeOH = 15:1 to 5:1) to obtain CA (24.1 mg). In addition, the fraction (392.2 mg) eluted with 100% MeOH was subjected to silica gel column chromatography (2.7 × 57 cm) by using a gradient elution (CH_2_Cl_2_:EtOAc = 15:1 to 5:1) to obtain BA (13.6 mg). The compounds’ identities were confirmed by TLC and NMR analysis.

### 4.3. Cell Culture and Viability Assay

RAW 264.7 macrophages were maintained at a density of 5 × 10^5^ cells/mL in Dulbecco’s modified Eagle’s medium supplemented with 10% heat-inactivated fetal bovine serum, penicillin G (100 units/mL), streptomycin (100 mg/mL), and L-glutamine (2 mM), and were incubated at 37 °C in a humidified atmosphere containing 5% CO_2_. The effect of the various experimental treatments on cell viability was evaluated by determining mitochondrial reductase function with an assay based on the reduction of MTT to formazan crystals. The formation of formazan is proportional to the number of functional mitochondria in the living cells. For the determination of cell viability, 50 µL MTT (2.5 mg/mL) was added to cell suspension (1 × 10^5^ cells/mL in each well of the 96-well plates) at a final concentration of 0.5 mg/mL, and the mixture was further incubated for 3–4 h at 37 °C. The formazan formed was dissolved in acidic 2-propanol, and the optical density was measured at 590 nm. The optical density of the formazan formed in the control (untreated) cells was considered as 100% viability.

### 4.4. Determination of Nitrite Production and PGE_2_, TNF-α, IL-1β, and IL-6 Assays

The production of nitrite, a stable end product of NO oxidation, was used as a measure of iNOS activity. The nitrite present in the conditioned medium was determined by using a method based on the Griess reaction. The concentrations of PGE_2_, TNF-α, IL-1β, and IL-6 in the culture medium were determined using ELISA kits (R&D Systems) according to the manufacturer’s instructions.

### 4.5. Preparation of Cytosolic and Nuclear Fractions

RAW 264.7 macrophages were homogenized in PER-Mammalian Protein Extraction Buffer (1:20, *w*/*v*) (Pierce Biotechnology, Rockford, IL, USA) containing freshly added protease inhibitor cocktail I (EMD Biosciences, San Diego, CA, USA) and 1 mM PMSF. The cytosolic fraction of the cells was prepared by centrifugation at 15,000× *g* for 10 min at 4 °C. Nuclear and cytoplasmic extracts were prepared using NE-PER nuclear and cytoplasmic extraction reagents (Pierce Biotechnology), respectively.

### 4.6. Western Blot Analysis

RAW 264.7 macrophages were harvested and pelleted by using centrifugation at 200× *g* for 3 min. Then, the cells were washed with phosphate-buffered saline and lysed in 20 mM Tris-HCl buffer (pH 7.4) containing a protease inhibitor mixture (0.1 mM phenylmethanesulfonyl fluoride, 5 mg/mL aprotinin, 5 mg/mL pepstatin A, and 1 mg/mL chymostatin). Protein concentration was determined using a Lowry protein assay kit (Sigma Chemical Co.). Thirty micrograms of protein from each sample were resolved by 12% sodium dodecyl sulfate-polyacrylamide gel electrophoresis, and then electrophoretically transferred onto a Hybond enhanced chemiluminescence nitrocellulose membrane (Bio-Rad, Hercules, CA, USA). The membrane was blocked with 5% skimmed milk and sequentially incubated with the primary antibody (Santa Cruz Biotechnology and Cell Signaling Technology) and a horseradish peroxidase-conjugated secondary antibody, and then subjected to enhanced chemiluminescence detection (Amersham Pharmacia Biotech, Piscataway, NJ, USA).

### 4.7. DNA-Binding Activity of NF-κB

The DNA-binding activity of NF-κB in nuclear extracts was measured using the TransAM kit (Active Motif, Carlsbad, CA, USA) according to the manufacturer’s instructions. Briefly, 30 μL of complete binding buffer (DTT, herring sperm DNA, and binding buffer AM3) was added to each well. The samples were nuclear extracts from RAW 264.7 macrophages stimulated for 30 min with LPS and treated with different concentrations of compounds. Then, 20 μL of the samples in the complete lysis buffer were added to each well (20 μg of nuclear extract diluted in complete lysis buffer). The plates were incubated for 1 h at room temperature with mild agitation (100 rpm on a rocking platform). After washing each well with wash buffer, 100 μL of diluted NF-κB antibody (1:1000 dilution in 1× antibody-binding buffer) was added to each well, and then the plates were incubated further for 1 h as before. After washing each well with the wash buffer, 100 μL of diluted HRP-conjugated antibody (1:1000 dilution in 1× antibody-binding buffer) was added to each well, followed by 1 h incubation as before. One hundred microliters of developing solution were added to each well for 5 min, followed by the addition of stop solution. Finally, the absorbance of each sample at 450 nm was determined by using a spectrophotometer within 5 min.

### 4.8. Transfection

Cells were transiently transfected with 50 nM of HO-1 siRNA and Nrf2 siRNA for 6 h using Lipofectamine 2000™ (Invitrogen), according to the manufacturer’s protocol, and recovered in fresh medium containing 10% fetal bovine serum for 24 h.

### 4.9. Statistical Analysis

Data were expressed as the mean ±SD of at least three independent experiments. To compare three or more groups, one-way analysis of variance followed by the Newman-Keuls post hoc test was used. Statistical analysis was performed by using GraphPad Prism software, version 3.03 (GraphPad Software Inc., San Diego, CA, USA).

## 5. Conclusions

In this study, two triterpenoid compounds, CA and BA, obtained from DKLE. BA and CA decreased pro-inflammatory mediators via inhibition of NF-κB pathways in LPS-stimulated RAW 264.7 macrophages. Moreover, only BA induced HO-1 induction via Nrf2 translocation, which was involved in their anti-inflammatory properties. These findings provided information on the mechanism of the anti-inflammatory actions of CA and BA from *D. kaki*. Additional studies on the biological effects of these compounds are warranted in the future.

## Figures and Tables

**Table 1:** ^1^H (400 MHz) and ^13^C (100 MHz) resonance assignment of coussaric acid (CA) (in pyridine-*d*_5_).

Carbon No.	δ ^13^C (ppm)	δ ^1^H (ppm)	Carbon No.	δ ^13^C (ppm)	δ ^1^H (ppm)
1	34.0	1.39–1.46 (m), 1.90 (m)	16	26.8	2.10–2.16 (m), 3.20–3.27 (m)
2	26.5	1.86 (m), 2.10–2.16 (m)	17	48.4	
3	70.0	4.45 (br s)	18	55.4	3.23 (br s)
4	44.0		19	73.0	5.74 (s)
5	50.2	1.95 (br s)	20	156.7	
6	19.2	1.56 (m), 1.72–1.77 (m)	21	29.0	2.45 (m), 3.12–3.27 (m), 3.16 (m)
7	34.3	1.41–1.48 (m), 1.72–1.77 (m)	22	39.5	2.10–2.16 (m), 2.30–2.34 (m)
8	40.4		23	23.6	1.62 (s)
9	47.8	2.10–2.16 (m)	24	65.7	2.45 (m), 3.16 (m)
10	37.5		25	16.1	0.99 (s)
11	24.3	2.07 (m), 2.10–2.16 (m)	26	17.2	1.10 (s)
12	128.3	5.63 (br s)	27	24.0	1.69 (s)
13	139.6		28	180.2	
14	42.2		29	27.6	1.64 (s)
15	29.2	1.34 (m), 2.30–2.34 (m)	30	105.3	4.80 (s), 5.00 (s)

**Table 2:** ^1^H (400 MHz) and ^13^C (100 MHz) resonance assignment of betulinic acid (BA) (in pyridine-*d*_5_).

Carbon No.	δ ^13^C (ppm)	δ ^1^H (ppm)	Carbon No.	δ ^13^C (ppm)	δ ^1^H (ppm)
1	39.1	1.01 (m), 1.68 (br s)	16	32.7	1.56 (m), 2.65 (m)
2	28.1	1.87 (m)	17	56.3	
3	78.1	3.47 (t, *J* = 7.2 Hz)	18	47.6	1.77 (br s)
4	39.4		19	49.5	3.55 (m)
5	55.7	0.82 (m)	20	150.7	
6	18.6	1.57 (m), 1.39 (m)	21	30.1	1.54 (m), 2.25 (m)
7	34.7	1.46 (m), 1.39 (m)	22	37.5	1.58 (m), 2.26 (m)
8	40.9		23	28.5	1.24 (s)
9	50.8	1.38 (m)	24	16.3	1.02 (s)
10	37.3		25	16.3	0.83 (s)
11	21.1	1.44 (m), 1.21 (m)	26	16.2	1.07 (s)
12	25.9	1.21 (m), 1.95 (m)	27	14.8	1.08 (s)
13	38.4	2.74 (m)	28	178.7	
14	42.4		29	110.3	4.96 (br s), 4.78 (s)
15	31.1	1.26 (m), 1.88 (m)	30	19.4	1.8 (s)
